# Harnessing the endosomal escape compartment as a strategy to enhance RNA delivery via lipid nanoparticles

**DOI:** 10.1016/j.omtn.2026.103018

**Published:** 2026-07-28

**Authors:** Tom Bourguignon, Preeti Sharma, Inbal Hazan-Halevy, Dan Peer

**Affiliations:** 1 Laboratory of Precision Nanomedicine, The Shmunis School of Biomedicine and Cancer Research, George S. Wise Faculty of Life Sciences, Tel Aviv University, Tel Aviv-Yafo, Israel; 2 Center for Nanoscience and Nanotechnology, Tel Aviv University, Tel Aviv-Yafo, Israel; 3 Department of Materials Science and Engineering, Tel Aviv University, Tel Aviv-Yafo, Israel; 4 Cancer Biology Research Center, Tel Aviv University, Tel Aviv-Yafo, Israel

## Main text

### Lipid nanoparticles and endosomal escape

Lipid nanoparticles (LNPs) have emerged as the leading platform for the delivery of RNA-based therapeutics.^1^ From the FDA approval of the first siRNA drug in 2018 (Onpattro) to the development of the first mRNA vaccines during the COVID-19 pandemic (Spikevax, Comirnaty), LNPs have overcome critical barriers to RNA delivery and accelerated the clinical translation of next-generation therapies. Yet, key challenges still hinder a global breakthrough in the market. At the systemic level, liver accumulation and off-target delivery represent fundamental issues; at the cellular level, endosomal escape remains a critical obstacle and has therefore been the focus of multiple publications in the field, including our recent review.^2^ Enhancing endosomal escape would improve RNA bioavailability and lower the administered doses for optimal effect.

LNP design is directly linked to the escape process.^3^ Following endocytosis, LNPs are entrapped within the endosome, whose maturation brings the intraluminal pH from ∼6.5 to ∼4.5. The mature endosome eventually fuses with the lysosome, where the RNA cargo is ultimately degraded. Therapeutic success thus depends on LNP ability to escape the endosomal pathway before reaching the lysosome. As the intraluminal pH decreases below the pKa of the LNP ionizable lipid, the latter becomes protonated and interacts with anionic lipids in the endosomal membrane. These electrostatic interactions induce a membrane structural transition from a lamellar to an inverted hexagonal phase, unstable, and promoting membrane disruption for successful escape to the cytosol. However, both foundational and current works report that less than 10% of LNPs manage to escape the endosome.^4^^,^^5^^,^^6^ Moreover, the precise characteristics of the escape compartment (the compartment within the endosomal pathway most prone to LNP escape) remain uncertain.

### The escape compartment as a therapeutic target

As highlighted in our review, numerous cutting-edge studies have focused on lipid engineering to maximize membrane instability and enhance endosomal escape. We believe that another approach, currently underexplored, would involve modulating cellular pathways themselves. If we could identify the escape compartment, preventing the endosome from maturing further might prolong LNP presence in this favorable stage and maximize therapeutic effects.

Along the endosomal pathway, several key stages are defined by the endocytic markers that characterize them: schematically, EEA1 and Rab5 for the early endosome, Rab11 for the recycling endosome, Rab7 for the late endosome, and LAMP1 for the lysosome.^7^ It is important to acknowledge the existence of a hybrid compartment bearing both Rab5 and Rab7, with the latter progressively replacing the former.

The main objective of our review is to compile and compare data from different publications to identify the escape compartment as precisely as possible, using endocytic markers as reference landmarks. We selected seven publications over the past thirteen years, from seminal works to the most recent ones, which offer a comprehensive and up-to-date view of the escape compartment when considered together. To characterize the area of interest, some works made use of highly valuable tools: galectins, notably Gal8 and Gal9. When the endosomal membrane is damaged by LNP escape and its luminal leaflet is exposed to the cytosol, galectins are recruited to the membrane. Thus, colocalization between Gal8/Gal9 and endocytic markers is a relevant indicator not only to identify the escape compartment, but also to relatively compare the efficiency of different LNP formulations.

**Figure 1:**
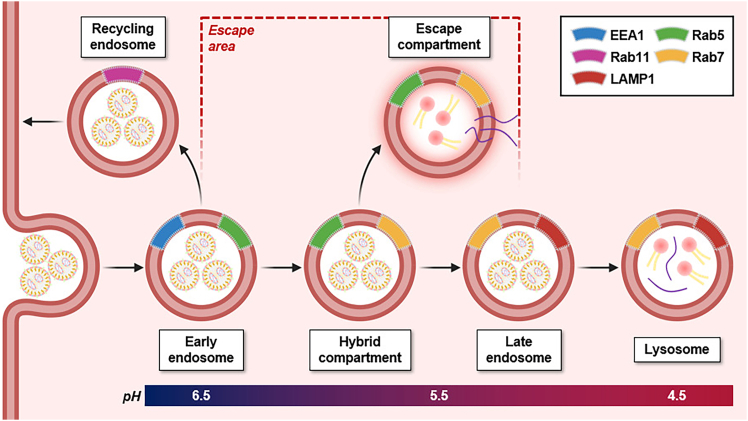
Schematic representation of LNP endosomal trafficking and the escape compartment As the endosome matures and ultimately fuses with the lysosome, the intraluminal pH progressively decreases from ∼6.5 to ∼4.5. Based on published data, endosomal escape is hypothesized to occur from a transient compartment characterized by the marker profile EEA1^−^/Rab5^+^/Rab11^−^/Rab7^±^/LAMP1^−^, indicating an intermediate stage of endosomal maturation. Created with BioRender.com.

Collectively, these seven publications suggest that the escape compartment might be characterized by the marker profile EEA1^−^/Rab5^+^/Rab11^−^/Rab7^±^/LAMP1^−^ (Figure 1). To successfully deliver their RNA cargo, LNPs must reach a stage of the pathway where the endosome has matured enough for the intraluminal pH to become lower than the ionizable lipid’s pKa, but not too much to prevent lysosomal degradation. Indeed, in their respective studies, Wittrup et al. and Herrera, Kim et al. reported Gal8 recruitment occurring both after EEA1 loss and before LAMP1 recruitment to the endosome.^5^^,^^8^ The role of Rab7 in the process is less certain, as Gal8 recruitment was reported both in its presence and in its absence.

These findings delineate the escape compartment more precisely, opening the door to new tools for LNP escape optimization. Inhibiting relevant pathways might entrap LNPs in this favorable area and enhance RNA delivery. Among the publications discussed in our review, two provide interesting data on this point. Cavegn et al. used inhibitors of the recycling endosome and reported an almost 2-fold increase in escape^9^; Sahay et al. silenced NPC1, a glycoprotein involved in recycling from late compartments, which resulted in a 4-fold increase in LNP efficiency.^10^ These two examples provide evidence that pathway silencing might be a relevant approach to increase cargo bioavailability.

### Model standardization

We also compare the experimental parameters used across publications (namely the ionizable lipid, LNP cargo, LNP size, included cell types and studied time points) and highlight the need for model standardization across the field to draw stronger conclusions from individual studies. Although every model has its own characteristics, we suggest standardizing several key parameters: including HeLa as a cell model, employing benchmark ionizable lipids such as ALC-0315 and SM-102, and focusing on a certain range of time points (1, 2 and 4 h) could harmonize findings in the field and help to build common strategies.

Endosomal escape remains one of the most challenging aspects of RNA delivery, and new tools are needed to further accelerate the clinical progress of the most cutting-edge therapies. In addition to lipid engineering, we propose escape compartment targeting as an alternative approach to maximize the outcomes of RNA-based therapeutics.

## Acknowledgments

This work was supported by the Harry Bloomfield international scholarship awarded to T.B. P.S. is the recipient of a fellowship from the Yoran Institute for Human Genome Research. D.P. receives funding support from the European Research Council (advanced grant 101055029) and Israel Science Foundation grant (2012/20 and 842/25).

## Declaration of interests

D.P. receives unrelated licensing fees (to patents on which he was an inventor) from, invested in, consults (or on scientific advisory boards or boards of directors) for, lectured (and received a fee), or conducts sponsored research at TAU for the following entities: ART Biosciences, BioNTech SE, Earli Inc., Kernal Biologics, LAND Therapeutics, Merck KGaA, New Phase Ltd., NeoVac Ltd., RiboX Therapeutics, Roche, SirTLabs Corporation, Teva Pharmaceuticals Inc.
